# Phenotypic resistance to pyrethroid associated to metabolic mechanism in *Vgsc*-L995F-resistant *Anopheles gambiae* malaria mosquitoes

**DOI:** 10.12688/wellcomeopenres.19126.1

**Published:** 2023-03-15

**Authors:** France-Paraudie A. Kouadio, Angèle N. Sika, Behi K. Fodjo, Christabelle G. Sadia, Sébastien K. Oyou, Allassane F. Ouattara, Chouaïbou S. Mouhamadou

**Affiliations:** 1 Environment and Health, Centre Suisse de Recherches Scientifiques en Côte d'Ivoire, Abidjan, 01 BP 1303 Abidjan 01, Cote d'Ivoire; 2 Natural Sciences, Université Nangui Abrogoua, Abidjan, 02 BP 801 Abidjan 02, Cote d'Ivoire

**Keywords:** Resistance selection, Anopheles gambiae, Deltamethrin, PermaNet 2.0, Tiassalé strain, P450 genes, Vgsc-L995F, Côte d’Ivoire

## Abstract

**Background: **The indiscriminate use of insecticides in agriculture and public health lead to a selection of resistance mechanisms in malaria vectors compromising vector control tools and strategies. This study investigated the metabolic response in the *Vgsc*-L995F *Anopheles* *gambiae* Tiassalé resistance strain after long-term exposure of larvae and adults to deltamethrin insecticide.

**Methods:** We exposed, over 20 generations, *Vgsc*-L995F *An. gambiae* Tiassalé strain larvae to deltamethrin (LS) and adults to PermaNet 2.0 (AS) and combining exposure at larvae and adult stages (LAS) and compared to unexposed (NS) group. All four groups were subjected to the standard World Health Organization (WHO) susceptibility tube tests using deltamethrin (0.05%), bendiocarb (0.1%) and malathion (5%). *Vgsc*-L995F/S *knockdown-resistance* ( *kdr)* mutation frequency was screened using multiplex assays based on Taqman real-time polymerase chain reaction (PCR) method. Additionally, expression levels of detoxification enzymes associated to pyrethroid resistance, including CYP4G16, CYP6M2, CYP6P1, CYP6P3, CYP6P4, CYP6Z1 and CYP9K1, and glutathione S-transferase GSTe2 were measured.

**Results: **Our results indicated that deltamethrin resistance was a response to insecticide selection pressure in LS, AS and LAS groups, while susceptibility was observed in NS group. The vectors showed varied mortality rates with bendiocarb and full susceptibility to malathion throughout the selection with LS, AS and LAS groups. *Vgsc*-L995F mutation stayed at high allelic frequency level in all groups with a frequency between 87% and 100%. Among the overexpressed genes, CYP6P4 gene was the most overexpressed in LS, AS and LAS groups.

**Conclusion:** Long-term exposure of larvae and adults of *Vgsc*-L995F resistant- *An. gambiae* Tiassalé strain to deltamethrin and PermaNet 2.0 net induced resistance to deltamethrin under a significant effect of cytochromes P450 detoxification enzymes. These outcomes highlight the necessity of investigating metabolic resistance mechanisms in the target population and not solely *kdr* resistance mechanisms prior the implementation of vector control strategies for a better impact.

## Introduction

Insecticides remain the key components of malaria vector control in Africa, used to treat bed nets and for indoor residual spraying (IRS). Intervention programs based notably on insecticide-treated nets (ITNs) have recently contributed to the reduction of malaria morbidity and mortality in sub-Saharan Africa by 62% between 2000 and 2019 ^1^. Unfortunately, the extensive use of insecticides in public health has subjected malaria vectors to enormous selective pressure, leading to the development of insecticide resistance, which leads to the loss of effectiveness of vector control tools ^2^. Many studies have reported vectors resistance to the main insecticide classes used in vector control programs ^3– 6^.

Insecticide resistance is described as the ability of insects to survive and adapt to the effects of an insecticide ^7^. Because of their knockdown effect for mosquitoes and low toxicity to humans, pyrethroids were the only recommended class of insecticides used in insecticide-treated nets (ITNs) until 2019 ^8^. Recently, several ITNs with new chemicals such as pyriproxyfen and Chlorfenapyr, pre-qualified by the World Health Organization (WHO) have been developed and are currently deployed in Africa ^8– 10^. However, although these ITNs still contain pyrethroid insecticides, their efficacy against resistant mosquitoes is reinforced by the presence of a second chemical, which is either a synergist or an insecticide.

In Africa, pyrethroid resistance has been linked mainly to two resistance mechanisms: target site resistance and metabolic resistance. Target site resistance involves non-synonymous mutations in the gene encoding the voltage-gated sodium channel ( *Vgsc*) present in the central nervous system of mosquitoes, which is a functional target of pyrethroid and DDT insecticides. They are often referred to as “knockdown resistance” ( *kdr*) because of the ability of insects possessing these alleles to be resistant to the insecticide’s knockdown effect. *Kdr* is conferred by two mutations occurring at the same position *Vsgc*-995F/S in the major African malaria vector *An. gambiae* ^11^. Both mutations have been reported to be widely distributed in *Anopheles* populations in sub-Saharan Africa ^12– 14^. The metabolic resistance is caused by the elevated level of detoxification enzyme activity and relates to metabolizing, which increases the metabolism or sequestration of the insecticide before it reaches its target site in mosquitoes ^15, 16^. Three major families of detoxification enzymes which are linked to insecticide resistance include cytochrome P450 (P450 or CYP) monooxygenases, carboxyl/choline esterases (CCEs), and glutathione- *S*-transferases (GSTs). Several studies have reported these detoxification enzymes are related to pyrethroid resistance in malaria vector ^2, 17, 18^. Other mechanisms of resistance involve cuticular thickening and overexpression of chemosensory protein ^19, 20^. The evolution of the pyrethroid resistance phenomenon and the multiple mechanisms of resistance represent a great challenge that requires attention.

In addition to the use of insecticides in public health, several studies have reported that the indiscriminate use of pyrethroids and other classes of insecticides for agricultural purposes has increased selection pressure for resistance in mosquito populations in Africa ^5, 6, 12, 21, 22^. Due to the growing population in Africa, the use of insecticides is becoming a key element in increasing agricultural yields ^23^. In West Africa, three countries including Côte d'Ivoire, Ghana, and Nigeria were the largest pesticide importer between 2005 and 2015 ^24^. Pyrethroids appeared to be the most popular insecticides used, accounting for 90% of all reported insecticides used in the southern part of the country. The use of insecticides in areas of intensive agriculture often results in the contamination of mosquito larvae breeding sites ^21^. The increasing use of pesticides in agricultural settings was also reported to influence insecticide resistance across Côte d'Ivoire. *Anopheles gambiae* larvae emerging from an irrigated rice field of Tiassalé were almost up to the insecticide classes used in public health. Therefore, environmental parameters, such as the presence of xenobiotic compounds, influence both the overall reaction of mosquitoes to pyrethroids and the selection of resistance mechanisms ^5, 21, 25, 26^.

Currently, the use of long-lasting insecticidal nets (LLINs) and pesticides for public health and agricultural purposes are incriminated as a source of resistance selection in mosquitoes, as it relies heavily on pyrethroid insecticides. However, the relative contribution of target site resistance and metabolic resistance mechanisms in the selection of pyrethroid resistance in *Anopheles* mosquitoes remains unclear. This study aimed to determine how much a pyrethroid phenotypic resistance can be driven by metabolic mechanism in *An. gambiae* mosquitoes. To reach this, we performed a resistance selection experiments with *Vgsc*-L995F resistance *An. gambiae* Tiassalé strain both at larvae and adult stages and determine underlying metabolic resistance mechanisms. We purposely used a high *Vgsc*-L995F-resistant *An. gambiae* colony to detect only an induced metabolic effect on mosquito phenotype.

## Methods

### Mosquito strain

This study was conducted with laboratory-reared colonies ( *An. gambiae* senso stricto specimens) isolated from the pyrethroid-resistant *An. gambiae* s.l mosquitoes collected in the irrigated rice field of Tiassalé, southern Côte d’Ivoire ^27^. The locality of Tiassalé is characterized by an intensive use of pyrethroid and neonicotinoid insecticides in agriculture ( *e.g.* irrigated rice) ^21, 28^. Earlier studies have shown that *An. gambiae* s.l from Tiassalé carry the *kdr* and *Ace1* point mutation as well as P450 genes conferring resistance to pyrethroids, DDT, carbamates and organophosphates ^13, 25, 26, 29^. The mosquito colony used in the present experiments derived from the *An. gambiae* Tiassalé strain maintained in the insectary of Centre Suisse de Recherche Scientifiques en Côte d'Ivoire (CSRS) over several generations since 2010, without being subjected to any insecticide selection pressure. Prior to start the selection the *Vgsc*-L995F allele mutation was confirmed in the Tiassalé *An. gambiae* strain using TaqMan RT-PCR method as described below.

### Laboratory resistance selection

The experiments used four colonies derived from the same parental population ( *i.e.*, G0 parent generation) of *An. gambiae* Tiassalé strain carrying *Vgsc*-L995F *kdr* mutation:

- Larvae-elected (LS) group: colony selected with deltamethrin at larval stage over 20 generations;
- Adult-Selected (AS) group: colony selected with PermaNet 2.0 LLIN at adult stage over 20 generations;
- Larvae+Adult Selected (LAS) group: colony selected at both larvae and adult stages with deltamethrin and PermaNet 2.0 respectively and
- Non-Selected (NS) group: colony non-subjected to any insecticide resistance selection, nor at larval stage and neither at adult stage, but reared over 20 generations.

The LS group mosquitoes were selected with a solution of deltamethrin insecticide prepared from a stock solution containing 100mg of deltamethrin (100%) powder in 1000 mL of water. We exposed only stage II larvae of each generation up to 20 generation for 24 hours to a sub-lethal dose of deltamethrin inducing 20% mortality (LD _20_). In order to minimize excessive losses while exerting resistance selection, we used the dose causing 20% mortality. To maintain the selection pressure, a new LD _20_ was determined after every five generations (G1, G6, G11, G16) with 100 larvae distributed per batch of 25 larvae and exposed for 24 hours in plastic cups containing 100 mL of water and fed with 0.075g powdered Friskies cat food. The different LD _20_ were determined by counting larvae survival and using PoloPlus 1.0 software (software using probit or logit regression analyses). Prior to each exposure, the deltamethrin solution was prepared 24 hours in advance to allow complete dilution of the deltamethrin powder in water. After exposure, all surviving larvae were transferred to clean water without insecticide, fed and allowed to emerge. At each generation, two- to five-day old female adult were blood-fed to obtain the next generation. We conducted the rearing at the CSRS insectary under standard conditions (26 °C ± 2 °C, 75% ± 10% and a photoperiod of 12:12 h).

In the AS group, the selection was conducted by exposing two- to five-dayold, non-blood fed adult female mosquitoes to new PermaNet 2.0 LLIN. PermaNet 2.0 LLIN was obtained from the National Malaria Control Program (NMCP). Permanent 2.0 LLIN is coated with a deltamethrin concentration of 55 mg/m ^2^. The long-term resistance selection was conducted by exposing adults of each generation to a Permanent 2.0 sub-lethal time causing 20% (LT _20_) mortality. To determine the LT _20_, we exposed four cohorts of 100 females at different set times in 15×15×15cm holding cages covered with Permanent 2.0 LLIN. After exposure, the mosquitoes were transferred to new cages covered with a non-treated net and the mortality was recorded 24 hours later. The LT _20_ was determined by counting the number of dead mosquitoes and using the PoloPlus software as describe above. We determined the LT _20_ before starting the experiment and at each series of five generations. At each generation, mosquitoes were all transferred to the same holding cage after exposure to LLIN and fed with sugar solution diluted at 10%. A total of 24 hours after exposure, mosquitoes were blood-fed in order to perform an insectary rearing. The AS group was reared in the CSRS insectary in the similar standard conditions (26°C ± 2°C, 75% ± 10% and a photoperiod of 12:12 h) described for LS group above.

The LAS group mosquitoes were selected by exposing larvae and adults to deltamethrin and PermaNet 2.0 LLIN as describe above.

The NS group was reared without any selection.

### Bioassays

To determine the phenotypic resistance within each selected group ( *i.e.*, LS, AS and LAS) or non-selected group ( *i.e.*, NS), susceptibility tube tests with discriminating doses of deltamethrin (0.05%), bendiocarb (0.1%) and malathion (5%) using treated papers according to WHO protocol ^7^ were performed. For each insecticide, four batches of 20–25 two- to five-day old female mosquitoes from the three selected groups and non-selected group were exposed to insecticide-impregnated papers in the presence of two negative control batches. The number of mosquitoes knocked out was recorded every five-minute intervals during the one-hour exposure. After exposure, tested mosquitoes were transferred into a holding tube and fed with 10% honey. Mortality was recorded 24 hours post-exposure. For each group, susceptibility assays were performed every fifth generation (G0, G5, G10, G15, G20).

### Mosquito sample preparation

After recording the 24-hour mortality, mosquitoes were killed by immersing them into absolute ethanol. They were later placed on filter papers to remove the extra ethanol before being transferred gently into a 1.5 mL microcentrifuge tube that contained RNAlater (Ambion, Inc., Austin, Texas, US). Then, we stored the mosquitoes at 4°C overnight to allow complete penetration of the product into the tissues. Exposed and negative control mosquitoes were stored separately. After one day, the excess RNAlater was removed and the tubes containing the mosquitoes were stored at -20°C until DNA and RNA extraction.

### DNA and RNA extraction

For each of the four mosquito groups, DNA and RNA extractions were performed with 30 individual mosquitoes randomly selected from the G0, G10 and G20 generations to detect mutation genes whereas for the detection of detoxification enzymes, the target genes expression levels were measured with 50 individuals split into pools of 10 mosquitoes for the G0 and G20 generations only per group (LS, AS, LAS and NS).

DNA and RNA were extracted using the MagnaMedics magnetic bead kit (MagnaMedics GmbH, Aachen, Germany). Both individual and batch mosquitoes were ground in 200 µL TE buffer (10 mM Tris-HCl, 1 mM EDTA, pH 8.0) per tube. Then 150 µL of lysis buffer was added, mixed for 15 s with a vortex before incubating for 10 min at room temperature with intermittent vortexing every 2 min for 15 s. After incubation, the mixture was spun by centrifugation at 16,000 ×g for 2 min. The supernatant was transferred to a new 1.5 mL tube, and then, 20 µL of magnetic beads and 440 µL of binding buffer were added and vortexed for 15 s. The mixture was incubated for 10 min as described above. To allow sedimentation of the magnetic beads, the tubes were placed on a magnetic rack for 2 min and the supernatant was discarded. The 200 µL of wash buffer was added twice to wash the beads while mixing and placed the tubes again for 2 min on the magnetic rack. Following the extraction, 180 µL of elution buffer to extract the nucleic acids were added and incubated for 10 min in a water bath at 50°C and vortexed in between as described above. After the incubation, the mixture was spun and placed on the magnetic rack. Finally, the supernatant that contained the purified DNA and RNA was transferred the new 1.5 mL tubes and kept at 80°C.

### Molecular analysis

The multiplex TaqMan Real Time – Polymerase Chain Reaction (RT-PCR) methods was used to detect two *kdr* point mutations from individual mosquitoes and detection of mutations in the voltage-dependent sodium channel target site ( *i.e.*, *Vgsc*-L995F/S) using the protocol developed by Bass *et al.* ^30^ as adapted by Mavridis *et al*. ^31 ^(see Table 1 for primers and probes sequences).

We measured the expression level of detoxification enzymes including CYP4G16, CYP6M2, CYP6P1, CYP6P3, CYP6P4, CYP6Z1, and CYP9K1, and the glutathione *S*-transferase GSTe2. For this step, we used the quantitative RT-PCR methods described by Mavridis *et al*. ^32^. We measured the expression levels of the detoxification enzymes as well as the reference gene coding for the ribosomal protein *S7* (RPS7). All RT-(qPCR) reactions were performed in 10 µL volumes including 1 µL of DNA/RNA, 9 µL of master mix comprising primers and probes. The final concentration of primers and probes is shown in Table 1. The reagents used in this study were provided by Fast-Track Diagnostics (FTD, Esch-sur-Alzette, Luxembourg). All reactions RT-PCR and RT-qPCR were performed in 96-well plates (Sarstedt, Nümbrecht, Germany; catalog number: 72.1980.202) on a C1000 touch CFX96 TM real-time PCR system (Bio-Rad Laboratories, Hercules, CA, US). The thermal cycler parameters were as follows; reverse transcription at 50°C for 15 min, RTase inactivation and initial denaturation for 3 min at 95°C, having 40 cycles of denaturation for 3 s at 95°C and extension step at 60°C for 30 s.

**Table 1.** List of primers and probes used for molecular analysis.

Reagent	Oligo_Name	Sequence (5' - 3')	Assay description Green: FAM Yellow: HEX Red: Atto647N		Optimized Concentration (nM)
Primer	kdr_F	CATTTTTCTTGGCCACTGTAGTGAT	kdr_Vgsc-L995F/S	none	500
Primer	kdr_R	CGATCTTGGTCCATGTTAATTTGCA	kdr_Vgsc-L995F/S	none	200
Probe	kdr-wt_P	CTTACGACTAAATTTC	wild type - kdr_Vgsc-L995	HEX	500
Probe	kdrW-mt_P	ACGACAAAATTTC	mutant - kdr-west_Vgsc-995F	FAM	500
Probe	kdrE-mt_P	ACGACTGAATTTC	mutant - kdr-east_Vgsc-995S	Atto647N	500
Primer	RPS7_Fj	CCACCATCGAACACAAAGTTGA	RPS7-Detox (A-D)	none	100
Primer	RPS7_R	TGCTGCAAACTTCGGCTATTC	RPS7-Detox (A-D)	none	200
Probe	RPS7_P1	CCGTGACGTTACGTTCGAATTCCCA	RPS7-Detox (A-D)	FAM	250
Primer	P3_Fj	ACAATGTGATTGACGAAACCCT	CYP6P3-Detox (A)	none	400
Primer	P3_R	GGATCACATGCTTTGTGCCG	CYP6P3-Detox (A)	none	500
Probe	P3_P1	ACCCGCGTACCGTCTGTGGACT	CYP6P3-Detox (A)	HEX	350
Primer	M2_F2	CTGGCGTTGAATCCAGAGGT	CYP6M2-Detox (A)	none	600
Primer	M2_Rj	GATACTTGCGCAGTGATTCATTAAG	CYP6M2-Detox (A)	none	400
Probe	M2_P	AGAGAAATCCTGCAAAAGCACAACGGAGA	CYP6M2-Detox (A)	Atto647N	250
Primer	K1_F	CCGACACGTGGTGATGGATAC	CYP9K1-Detox (B)	none	200
Primer	K1_Rj	CGTCGTCGGTCCAGTCAAC	CYP9K1-Detox (B)	none	400
Probe	K1_P	CAATCTTCTGATGCAGGCCCGCAA	CYP9K1-Detox (B)	HEX	300
Primer	P4_Fj	CTGGACAACGTTATCAATGAAACC	CYP6P4-Detox (B)	none	400
Primer	P4_R	GCACGGTGTAATCACGCATC	CYP6P4-Detox (B)	none	500
Probe	P4_P	CCGATCGAGTCACTTTCGCGCG	CYP6P4-Detox (B)	Atto647N	300
Primer	Z1_Fj	CCCGCAACTGTATCGGTCTG	CYP6Z1-Detox (C)	none	100
Primer	Z1_R	TTCGGTGCCAGTGTGATTGA	CYP6Z1-Detox (C)	none	600
Probe	Z1_P1	TGATGCTGTCCCGATTTAACTTTTCGGC	CYP6Z1-Detox (C)	HEX	250
Primer	TE2_Fj	CCGGAATTTGTGAAGCTAAACC	GSTE2-Detox (C)	none	100
Primer	TE2_R	GCTTGACGGGGTCTTTCGG	GSTE2-Detox (C)	none	400
Probe	TE2_P	CGGTACGATCATCACCGAGAGCCAC	GSTE2-Detox (C)	Atto647N	300
Primer	P1_Fj	ACAGGTGGTGAACGAAACCC	CYP6P1-Detox (D)	none	100
Primer	P1_R	GGTGTAATCCTGTCCCGCAA	CYP6P1-Detox (D)	none	500
Probe	P1_P	CCGCTCGAAACGACGCTGCG	CYP6P1-Detox (D)	HEX	300
Primer	G16_Fj	GTCCAAGAAGTTGCGTCGGAC	CYP4G16-Detox (D)	none	200
Primer	G16_R	TCTTCGATTTGCGTTGACGTG	CYP4G16-Detox (D)	none	200
Probe	G16_P1	CTGCAGGCCGACATCATTTTGAAGC	CYP4G16-Detox (D)	Atto647N	300

### Statistical analysis

Mortality results during susceptibility tests were interpreted according to WHO criteria ^7^: a mortality below 90% suggests resistance, equal to or greater than 98% indicates susceptible; and between 90% and 97% suggests a possibility of resistance that needs to be confirmed. We performed the survival analysis to visualize the time of pyrethroid knockdown by comparing the LS, AS, LAS and NS groups to the parent population (G0).

The expression level of genes determined at G0, G10 and G20 for LS, AS, LAS and NS groups were analyzed using the method of Pfaffl ^33^ implemented the genetic analysis software REST 2009 version v2.013. This software allowed to enter the Threshold Cycle (C _T_) values of the genes detected at G0, G10 and G20 generations. We calculated the fold change of each gene of interest relative to the parental G0 generation with 95% confidence intervals and P-values within each group. The C _T_ values of the reference gene coding for the ribosomal protein (RPS7) and of each target gene for each generation were re-assigned jointly to G10 and G20 (with or without insecticide selection) and G0 which represented the parent population. The fold changes were calculated based on the average values after 2,000 iterations.

Statistical analyses were performed with R software version 4.0.3 ^34^ using RStudio version 1.3.1093 ^35^. The R package tidyverse was used for data sorting, manipulation and visualization. We used the R packages survival and survminer to plot the survival curves and calculated the Kaplan-Meier estimates ^36^ with as significance level set at α = 0.05. We conducted a Chi-square test to compare the significance level of the mortalities between each of the three LS, AS and LAS and groups and NS group.

## Results

### Knockdown time

**Figure 1.**
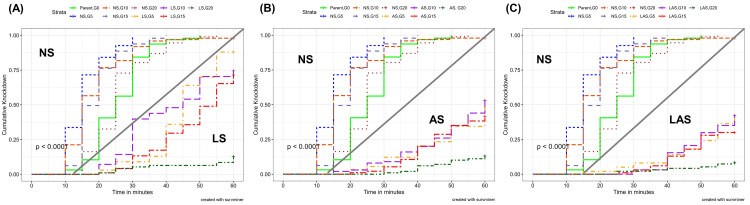
Kaplan-Meier survival curves showing the cumulative survival over the 60 minutes exposure to diagnostic concentrations of deltamethrin (0.05%) in the WHO insecticide susceptibility assay. The P-values represent the Log-rank test p-value. ( **A**) NS-LS; ( **B**); NS-AS; ( **C**) NS-LAS.

The knockdown effects varied substantially across generations within the LS, AS, LAS and NS groups ( Figure 1).

The knockdown rate observed in LS group ( Figure 1A) ranged from 13% to 100%. The highest rate was obtained at G0 (parent population) after 50 min while the lowest was found at the end of the one-hour exposure time with G20 adults. The insecticide knocked down more than 50% of the parent mosquitoes (G0) after 25 min of exposure. After five generation of selection, time allowing 50% knockdown was estimated at 45 min before reaching a maximum kdt _50_ of 55 min at G15. However, it was not possible to determine kdt _50_ for G20 because corresponding knockdown rates were less than 15% at any time point during exposure.

In the AS group ( Figure 1B), the knockdown rate declined from 100% (G0) to 13% at G20 as observed in the LS group. However, kdt _50_ was estimated at 60 min and observed early after five generations of selection while it was not determined there from G15.

In the LAS group ( Figure 1C), the knockdown rate dropped to 8% at G20 while it was 100% at G0. However, no kdt _50 _was determined at any generation except at G0.

In the NS group, the knockdown rate was kept very high for all generations comparing to LS, AS, and LAS ( Figure 1A, B, C). The kdt _50 _was recorded much earlier (between 15 and 25 min) during the exposure without substantial variations among the generations.

### Change in mosquito mortality and susceptibility to pyrethroid during the selection

**Figure 2.**
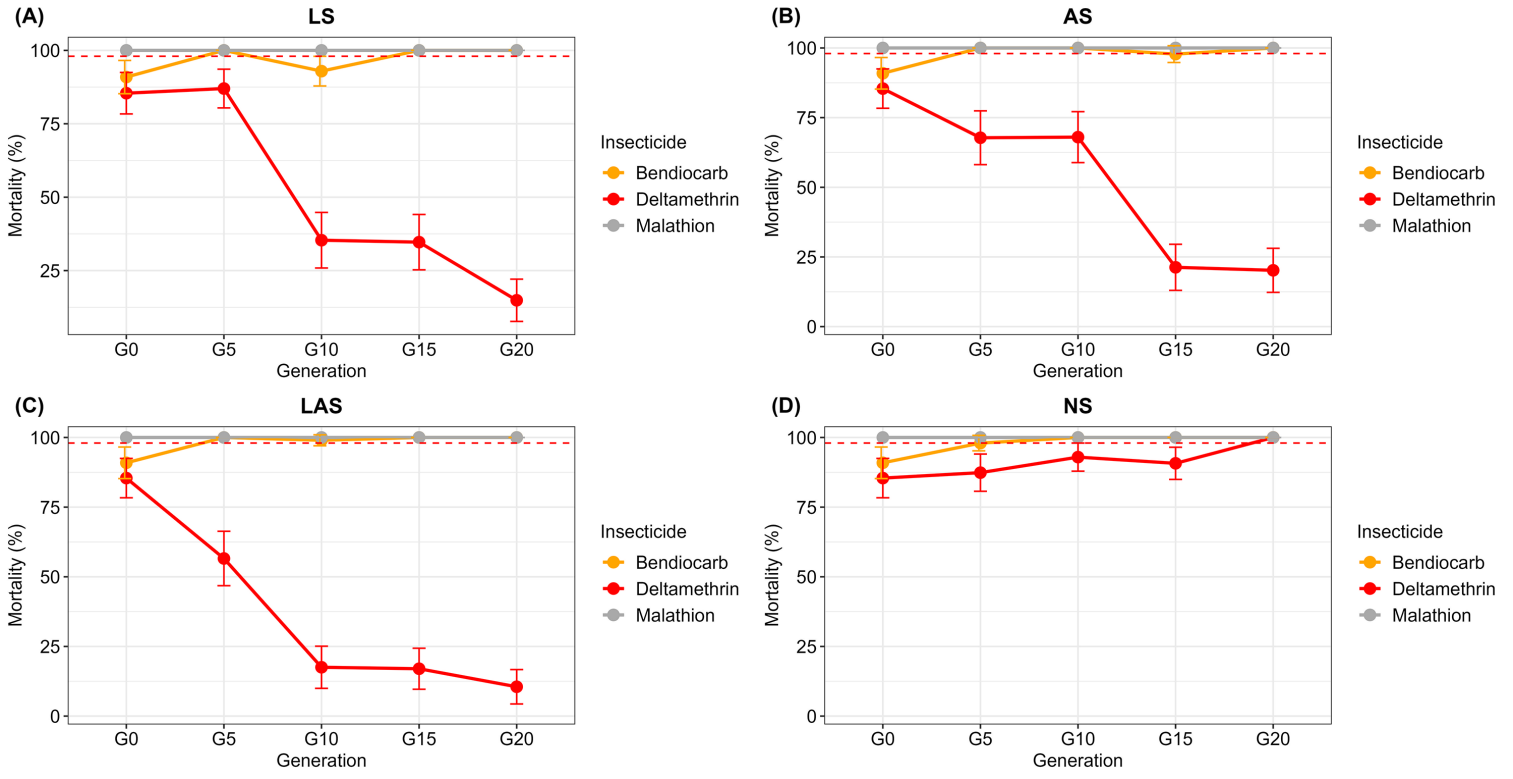
**Dynamics of mortality with bendiocarb (0.1%), deltamethrin (0.05), malathion (5%) in *An. gambiae* Tiassalé divided into three groups (A) LS, (B) AS, (C) LAS and (D) NS.** Error bars indicate 95% confidence intervals. The mortality threshold of 98% indicates full susceptibility according to WHO criteria ^7^.

Mortalities observed in controls were less than 5% for each WHO tube test performed, confirming the validity of the test. Figure 2 shows the WHO susceptibility test data in LS, AS, LAS and NS groups during the selection process. Mortality with deltamethrin, which was 85% in parent population G0, considerably varied among LS, AS, LAS and NS groups and across generations.

Indeed, in the selected LS, AS and LAS groups ( Figure 2A, B, C), deltamethrin mortality dropped significantly from G0 to G20 (P < 0.001). Within LS group, deltamethrin mortality rate decreased significantly (X ^2^ = 94.5; df =1; P < 0.001) to 14.9% at G20 with a resistance factor of 5.7 ( Figure 2A). The mortality in LS group started to differ significantly (X ^2^ = 77.3; df = 1; P < 0.001) after 10 generations. Additionally, mortality decreased significantly (X ^2^ = 83.1; df = 1; P < 0.001) to 20.2% at G20 in AS group ( Figure 2B) with a resistance factor of 2.1 and a significant difference (X ^2^ = 8.1; df = 1; P < 0.01) observed just after five generations. The greatest resistance factor (8.1%) was found in the LAS group with mortality declining to 10.9% at G20 ( Figure 2C). A significant difference (X ^2^ = 19.6; df = 1; P < 0.001) was observed as early as G5.

In the NS group ( Figure 2C), deltamethrin mortality increased progressively, and reached a rate of 100% at G20 ( Figure 2D). Deltamethrin mortality in NS group was significantly higher compared with the three LS group (X ^2^ = 137.4; df = 1; P < 0.001), AS group (X ^2^ = 125.2, df = 1, P < 0.001) and LAS group (X ^2^ =150.9, df = 1, P < 0.001).

Overall, very high mortalities (100%) were observed in the LS, AS, LAS and NS groups at any generations ( *i.e.*, from G0 to G20) with both bendiocarb and malathion, thus suggesting full susceptibility of all the four mosquito groups to both insecticides ( Figure 2A, B, C, D).

### Allelic frequency

**Figure 3.**
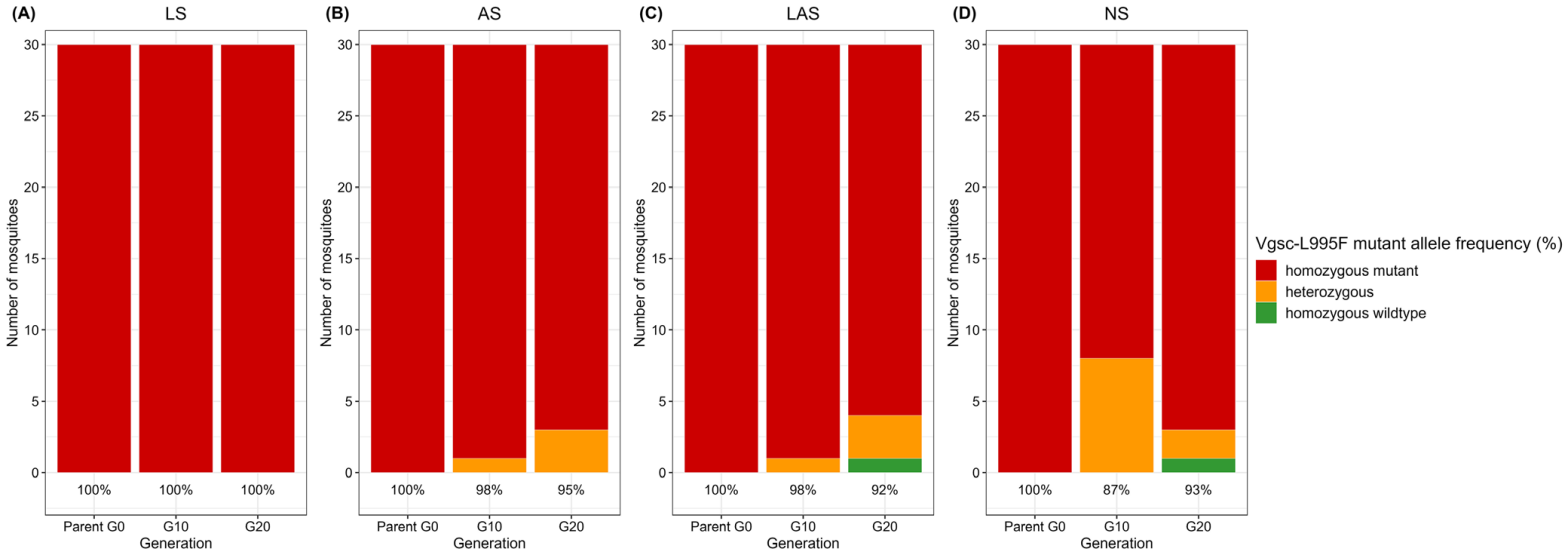
Dynamics of target site mutation *Vgsc*-L995F through resistance selection. ( **A**) LS group; ( **B**) AS group; ( **C**) LAS group and ( **D**) NS group. Percentages indicate the frequency of resistance alleles while bars indicate actual numbers. For each group, the *Vgsc*-L995F mutation was genotyped in individual mosquitoes.

The target site resistance gene *Vgsc*-L995F remained detectable at a high allelic frequency in the three selected groups (LS, AS, and LAS) as well as in non-selected groups (NS) ( Figure 3). However, the mutant *Vgsc*-L995S allele was not found in any groups. *Vgsc*-L995F allelic frequency remained the same from G0 to G20 in the LS group ( Figure 3A), but there was a slight decrease in the frequencies observed in the AS ( Figure 3A), LAS ( Figure 3B) and NS ( Figure 3C) groups. In the NS group, the frequency of *Vgsc*-L995F declined from 100% at G0 to 85% at G10, before rising to 93% at G20.

### Metabolic enzyme

**Figure 4.**
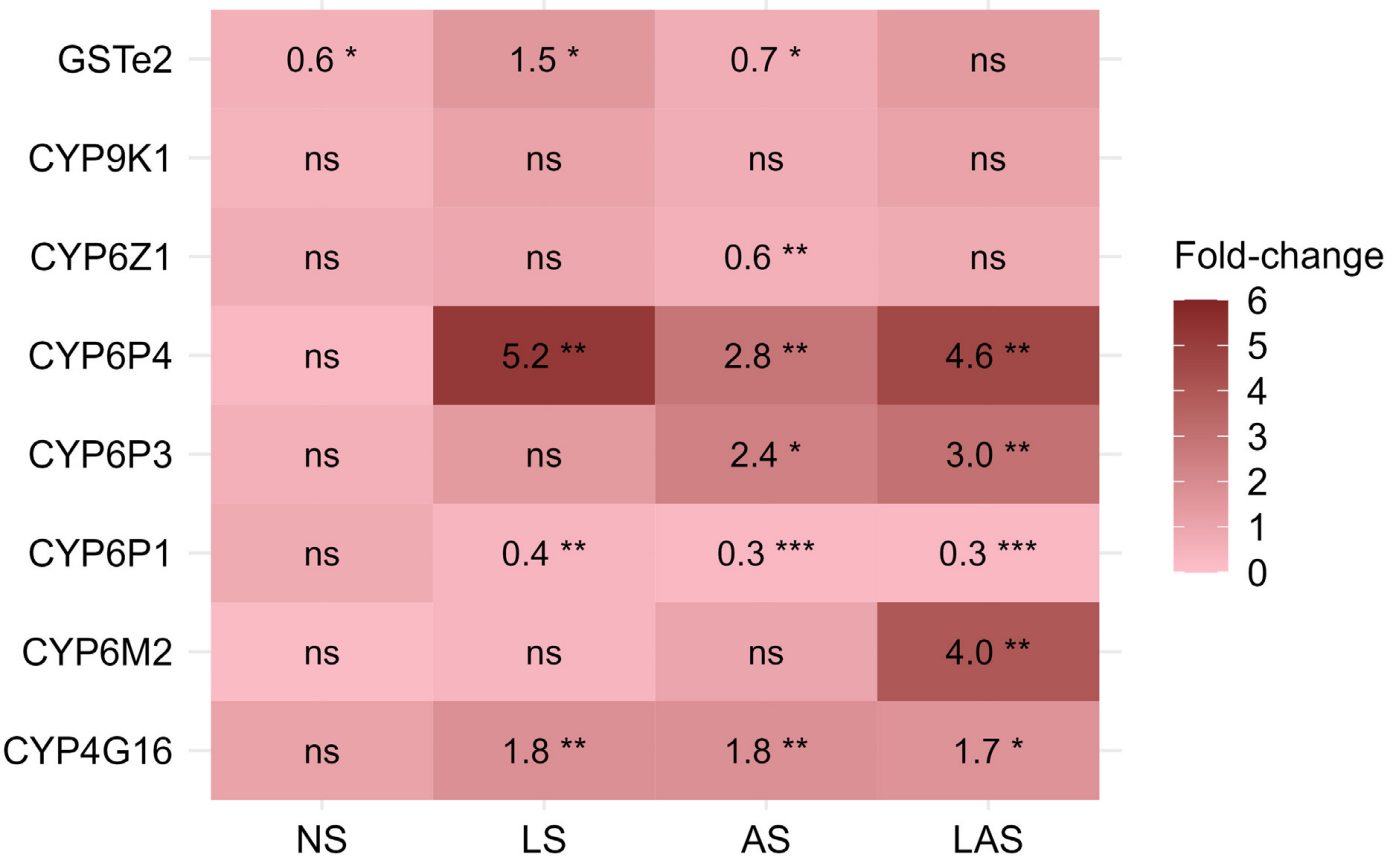
Differential expression levels for putative detoxifying genes measured in selected groups. Fold changes give the change in expression level of mosquito populations at the 20th generation relative to the reference generation (parent G0). Fold changes were estimated using REST2009 analysis software for each gene. ns: not significant; * p < 0.05; ** p < 0.01; *** p < 0.001.

Among the eight detoxification genes, the CYP6P4, CYP4G16, and glutathione *S*-transferase (GSTe2) were found to be overexpressed in LS group, while the CYP6P3, CYP6P4 and CYP4G16 were found overexpressed in AS group and the CYP6P3, CYP6P4, CYP6M2 and CYP4G16 were overexpressed in LAS group, when comparing G20 to G0 ( Figure 4). In contrast, none of the eight genes were overexpressed in the NS group at G20. The CYP6P4 was the most overexpressed gene with 5.2-fold, 2.8-fold and 4.6-fold higher up at G20 compared to the G0 parent in the three selected LS, AS and LAS groups respectively. The CYP4G16 gene, responsible for cuticular resistance, was detected, up-regulated about two-fold higher at G20 compared with G0 (P < 0.01) in the three selected groups. In the AS group, we found CYP6P3 gene significantly overexpressed with an up-regulation up to 2.4-fold compared to G0 (P < 0.05). The glutathione *S*-transferase GSTe2 was overexpressed about 1.5-fold higher in the LS group at G20 in comparison with G0 (P < 0.05). CYP6M2 gene associated to pyrethroid resistance was found as the second gene significantly overexpressed with 4-fold higher at G20 compared to G0 in LAS group.

## Discussion

The current study assessed the dynamics of insecticide resistance mechanism across different generations of *An. gambiae* carrying *Vgsc*-L995F exposed to deltamethrin. The outcomes showed that repeated exposure of *An. gambiae* larvae and adults to deltamethrin maintained the resistance mutation *Vgsc*-L995F allele at a high level, alongside the selection for detoxification genes. This selection of metabolic resistance genes was associated with pyrethroid resistance that induced a significant reduction in the susceptibility and mortality of *An. gambiae* Tiassalé strain exposed to deltamethrin across generations.

In fact, the phenotypic resistance, associated with a drastic reduction in mosquito mortality, increased significantly after only a few generations during selection either at larval or adult stages, and both at larval and adult stages concomitantly (G5) compared to non-selected group. This suggests that the increase in mosquitoes’ resistance is results from selection pressure exerted by repeated exposure to a chemical insecticide either directly in their environment at larval stage or adult stage through contact with a treated surface such as bed net across generations. Similarly, recent laboratory studies have shown that *An. coluzzii* mosquitoes gain resistance when subjected to continuous selection pressure but loose this resistance in the absence of insecticide exposure after 15 generations ^37^.

 The frequency of *Vgsc*-L995F allele stayed at a very high level in the selected and non-selected groups throughout the selection, suggesting the increased level of phenotypic resistance to deltamethrin in our context is due to the involvement of metabolic resistance as shown the overexpression of some detoxification genes. Earlier studies in *An. gambiae* and *Culex pippiens* mosquitoes ^37, 38^ reported that in a population where the *kdr* mutation is fixed or absent, phenotypic resistance could be associated with a metabolic resistance mechanism. On the other hand, the phenotypic resistance is lost if the selection pressure is not maintained; this was the case in our non-selected group where susceptibility test did not reveal any apparent resistance. Our data are consistent with studies conducted on *Aedes aegypti* in which significant loss of resistance was observed in the absence of selection pressure over 10 generations ^39^. These results also suggest that under field conditions, resistance can decline without insecticide pressure, likely due to the fitness costs associated with insecticide resistance.

In response to resistance selection with deltamethrin directly by exposure of larvae to deltamethrin or adults through PermaNet 2.0 LLIN or by combined exposure (larvae and adult stages), the P450 enzymes including CYP6M3, CYP6M2, CYP6P4, CYP4G16 and the glutathione *S*-transferase GSTe2 were overexpressed. The overexpression of four P450 genes confirms their roles in insecticide detoxification as they have previously been associated with pyrethroid resistance in *Anopheles* species ^40^, as reprorted in Tiassalé 13 strain ^41^ and in *An. arabiensis* from Ethiopia, including the GSTe2 ^42^. Indeed, in the present experiments, detoxification enzyme activity increased significantly in the LS, AS and LAS groups after 20 generations of selection from the parent G0 individuals having the *kdr*-L995F mutation. In contrast, no increase in detoxification gene activity was observed in the NS group. This observation could suggest that the metabolic resistance has strengthened under increasing selection pressure and played an important role in the development of high levels of resistance. A similar study examining pyrethroid resistance selection in *Culex pippiens pallens* mosquitoes found that the *kdr* mutation and metabolic genes contribute to pyrethroid resistance but play different roles under low and high selection pressure ^38^.

The CYP6P4 gene was the most overexpressed enzyme in LS, AS and LAS groups. The fact that this gene was the most overexpressed might imply that it plays a key role in the metabolic resistance against deltamethrin. This cytochrome P450 was detected in wild pyrethroid-resistant *An. coluzzii* mosquito populations collected in irrigated rice fields treated with pesticides ( *e.g.*, pyrethroid, organophosphate, triazine, carbamate, neonicotinoid) in Tiassalé and southern region of Côte d'Ivoire ^26, 43^. Another earlier and previous study has demonstrated that CYP6P4 is the major P450 involved in pyrethroid resistance in *kdr*-free *An. arabiensis* in Chad ^17^. Therefore, further studies are needed to better understand the role of CYP6P4 in deltamethrin metabolism in the *An. gambiae*. In addition, CYP6P3 and CYP6M2, found to be associated with pyrethroid resistance ^44, 45^ and CYP4G16 involves in cuticular resistance ^46^, were significantly overexpressed in LS, AS and LAS groups respectively. This could explain the low mosquito mortality to deltamethrin in the LS, AS and LAS groups observed in the present laboratory studies. Similarly, a recent laboratory selection study detected overexpression of these enzymes in the laboratory-reared resistant strain *An. gambiae* Tiassalé 13 and VK7 2014 strains subjected to selection pressure with WHO paper impregnated with deltamethrin 0.05% ^41^. Moreover, GSTe2 was found as an overexpressed detoxification gene in *An. gambiae* Tiassalé 13 ^41^, in *An. funestus* mosquitoes from Benin ^44^ and *An. arabiensis* from Ethiopia ^42^.

 Our current experiment showed that repeated exposure of laboratory colony of *An. gambiae* Tiassalé strain larvae to deltamethrin and adult to PermaNet 2.0 LLIN or combining exposure at both larvae and adult stages create pyrethroid resistance and select for P450 detoxification enzymes. The selection of metabolic resistance genes and increase in phenotype resistance in *kdr* resistance- *An. gambiae*, highlight the importance of metabolic resistance mechanism in the overall resistance phenomena. This suggests in our case that when selection pressure is high upon exposure to pyrethroids, mosquitoes with metabolic resistance background are the most selected. Wide use of insecticides ( *e.g.*, deltamethrin) in agriculture for crop protection and pyrethroid-treated vector control tools in public health are seriously threatening the efficacy of existing vector control programmers by inducing and spreading metabolic resistance in local mosquito vectors. Therefore, there is a need to integrate new classes of insecticides (neonicotinoids, pyrroles) into malaria vector control strategies to delay or stop the spread of pyrethroid resistance. Based on this study, it would be advisable for vector control strategies to extend beyond the search for *kdr* mutation only, as this does not appear to play more important role than metabolic resistance. Characterization of metabolic resistance should be an integrated part of insecticide resistance investigation prior to a vector control strategy implementation.

## Conclusions

Repeated exposure of *kdr* resistant- *An. gambiae* Tiassalé strain as larvae to a sublethal dose of deltamethrin and adults to PermaNet 2.0 LLIN and combining exposure over 20 generations in laboratory demonstrated an induction of very high level of resistance to deltamethrin triggered by the cytochromes P450 detoxification enzymes. Additionally, during the experiments, increased occurrences of these genes was proportionally associated with significant reductions in susceptibility and mortality of exposed groups against deltamethrin. However, the *kdr Vgsc*-L995F frequency remained very high from parental generation to the end of the experiment. Prospective studies to implement control strategies must consider investigating metabolic resistance mechanisms in the target population and not solely *kdr* resistance mechanisms.

## Data Availability

Zenodo. Phenotypic resistance to pyrethroid associated to metabolic mechanism in Vgsc-L995F resistant-Anopheles gambiae malaria mosquitoes. DOI: https://doi.org/10.5281/zenodo.7669513 ^47^ Data are available under the terms of Creative Commons Zero “No rights reserved” data waiver (CC-BY 4.0 Public domain dedication)
