# Supplementary material for: Phenotypic resistance to pyrethroid associated to metabolic mechanism in Vgsc-L995F-resistant Anopheles gambiae malaria mosquitoes
Source: Wellcome Open Res. 2023 Jun 27;8:118. Originally published 2023 Mar 15. [Version 2] doi: 10.12688/wellcomeopenres.19126.2 (PMC10308139; doi:10.12688/wellcomeopenres.19126.2)
Supplement: Peer review history

# Peer review: Reviewer response for version 2

**DOI:** 10.21956/wellcomeopenres.21732.r61432

**Published:** 2023-07-03

**Authors:** Salum Azizi

**Affiliations:** 1 Kilimanjaro Christian Medical University College, Moshi, Tanzania

I am happy that all issues that I have identified are addressed by authors in the new revised manuscript.

Is the work clearly and accurately presented and does it cite the current literature?

Yes

If applicable, is the statistical analysis and its interpretation appropriate?

Yes

Are all the source data underlying the results available to ensure full reproducibility?

Yes

Is the study design appropriate and is the work technically sound?

Yes

Are the conclusions drawn adequately supported by the results?

Yes

Are sufficient details of methods and analysis provided to allow replication by others?

Partly

Reviewer Expertise:

My area of research is on; efficacy testing of mosquito control tools, mosquito-borne disease control

I confirm that I have read this submission and believe that I have an appropriate level of expertise to confirm that it is of an acceptable scientific standard.

# Peer review: Reviewer response for version 2

**DOI:** 10.21956/wellcomeopenres.21732.r61433

**Published:** 2023-06-28

**Authors:** Arthur Sovi

**Affiliations:** 1 Centre de Recherche Entomologique de Cotonou, Cotonou, Benin

My comments are taken into account.

Is the work clearly and accurately presented and does it cite the current literature?

Yes

If applicable, is the statistical analysis and its interpretation appropriate?

I cannot comment. A qualified statistician is required.

Are all the source data underlying the results available to ensure full reproducibility?

Yes

Is the study design appropriate and is the work technically sound?

Yes

Are the conclusions drawn adequately supported by the results?

Yes

Are sufficient details of methods and analysis provided to allow replication by others?

Yes

Reviewer Expertise:

Medical Entomology

I confirm that I have read this submission and believe that I have an appropriate level of expertise to confirm that it is of an acceptable scientific standard.

# Peer review: Reviewer response for version 1

**DOI:** 10.21956/wellcomeopenres.21203.r59613

**Published:** 2023-06-19

**Authors:** Arthur Sovi

**Affiliations:** 1 Centre de Recherche Entomologique de Cotonou, Cotonou, Benin

The present paper is interesting as it investigates how much a pyrethroid phenotypic resistance can be driven by metabolic mechanism in *An. gambiae* s.l. mosquitoes. The methodology used is clearly described and a fair conclusion was drawn based on the results gathered.

 Nevertheless, I have the below minor recommendations/comments for the authors:

1. Introduction, first paragraph, line 4: Replace ITNs by LLINs. Also, make sure to do that throughout the manuscript.
2. Introduction, end of fourth paragraph: I’m not sure to figure out what you mean through the following sentence: “ *Anopheles gambiae* larvae emerging from an irrigated rice field of Tiassalé were almost up to the insecticide classes used in public health.” You need to reword it to make it understandable.
3. When it is the complex, please make sure to mention *An. gambiae* s.l., not *An. gambiae*. Please correct it throughout the manuscript.
4. There are several P450 genes, on what basis did you choose those (CYP4G16, CYP6M2, CYP6P1, CYP6P3, CYP6P4, CYP6Z1 and CYP9K1) whose level of expression you evaluated in your work?
5. Discussion: This suggests that the increase in mosquitoes’ resistance **is** results from selection pressure exerted by repeated exposure to a chemical insecticide either directly in their environment at larval stage or adult stage through contact with a treated surface such as bed net across generations. The “is” should be deleted.
6. Discussion: “pippiens” should be “pipiens”.
7. Discussion: The overexpression of four P450 genes confirms their roles in insecticide detoxification as they have previously been associated with pyrethroid resistance in Anopheles species40, as **reprorted **in Tiassalé 13 strain. “reprorted” should be “reported”.
8. Are there any specific reasons for which the three P450s genes were not overexpressed?
9. Discussion: Wide use of insecticides (e.g., deltamethrin) in agriculture for crop protection and pyrethroid-treated vector control tools in public health are seriously threatening the efficacy of existing vector control **programmers **by inducing and spreading metabolic resistance in local mosquito vectors. “programmers” should be “programme” **.**
10. 11-Discussion: Therefore, there is a need to integrate new classes of insecticides (neonicotinoids, pyrroles) into malaria vector control strategies to delay **or stop** the spread of pyrethroid resistance. I would suggest to remove the word “stop”.

Is the work clearly and accurately presented and does it cite the current literature?

Yes

If applicable, is the statistical analysis and its interpretation appropriate?

I cannot comment. A qualified statistician is required.

Are all the source data underlying the results available to ensure full reproducibility?

Yes

Is the study design appropriate and is the work technically sound?

Yes

Are the conclusions drawn adequately supported by the results?

Yes

Are sufficient details of methods and analysis provided to allow replication by others?

Yes

Reviewer Expertise:

Medical Entomology

I confirm that I have read this submission and believe that I have an appropriate level of expertise to confirm that it is of an acceptable scientific standard, however I have significant reservations, as outlined above.

# Response

**Published:** 2023-06-20

**Authors:** France-Paraudie Kouadio

**Affiliations:** Centre Suisse de Recherches Scientifiques en Côte d'Ivoire, Cote d'Ivoire

1. Introduction, first paragraph, line 4: Replace ITNs by LLINs. Also, make sure to do that throughout the manuscript. **Done**
2. Introduction, end of fourth paragraph: I’m not sure to figure out what you mean through the following sentence: “ *Anopheles gambiae* larvae emerging from an irrigated rice field of Tiassalé were almost up to the insecticide classes used in public health.” You need to reword it to make it understandable. **Done**
3. When it is the complex, please make sure to mention *An. gambiae* s.l., not *An. gambiae*. Please correct it throughout the manuscript. **Done**, **we want to specify that the strain used here is a population of *An. gambiae***
4. There are several P450 genes, on what basis did you choose those (CYP4G16, CYP6M2, CYP6P1, CYP6P3, CYP6P4, CYP6Z1 and CYP9K1) whose level of expression you evaluated in your work?
**As our work was a resistance selection study with the pyrethroid insecticide deltamethrin, we naturally chose the genes that metabolize pyrethroids, including, the major pyrethroid metabolizers (CYP6M2, CYP6P3 and CYP9K1).**
5. Discussion: This suggests that the increase in mosquitoes’ resistance **is** results from selection pressure exerted by repeated exposure to a chemical insecticide either directly in their environment at larval stage or adult stage through contact with a treated surface such as bed net across generations. The “is” should be deleted. **Done**
6. Discussion: “pippiens” should be “pipiens”. **Done**
7. Discussion: The overexpression of four P450 genes confirms their roles in insecticide detoxification as they have previously been associated with pyrethroid resistance in Anopheles species40, as **reprorted **in Tiassalé 13 strain. “reprorted” should be “reported”. **Done**
8. Are there any specific reasons for which the three P450s genes were not overexpressed?
**The three non-overexpressed P450 genes probably metabolize pyrethroid insecticides other than the deltamethrin used in our study. Conversely, CYP6Z1, one of these three genes, has been shown in several studies to metabolize DDT, suggesting why it was not overexpressed in our case.**
9. Discussion: Wide use of insecticides (e.g., deltamethrin) in agriculture for crop protection and pyrethroid-treated vector control tools in public health are seriously threatening the efficacy of existing vector control **programmers **by inducing and spreading metabolic resistance in local mosquito vectors. “programmers” should be “programme” **. Done**
10. 11-Discussion: Therefore, there is a need to integrate new classes of insecticides (neonicotinoids, pyrroles) into malaria vector control strategies to delay **or stop** the spread of pyrethroid resistance. I would suggest to remove the word “stop”. **Done**

# Peer review: Reviewer response for version 1

**DOI:** 10.21956/wellcomeopenres.21203.r59625

**Published:** 2023-06-19

**Authors:** Salum Azizi

**Affiliations:** 1 Kilimanjaro Christian Medical University College, Moshi, Tanzania

This work highlights the importance of surveying metabolic resistance instead of limiting it to kdr. This work has been able to link metabolic resistance to lowered mortality. The authors has clearly stipulated the negative consequences to malaria vector control if this is left unchecked. The findings from the study are very well presented.  

 The results show clear role of selection in all groups (LS, AS, LAS) in P450 genes overexpression and its effect on a reduced susceptibility to mosquitoes. The authors have also indicated that the kdr was almost fixed and had no obvious role in the resistance. This work is very relevant in endemic countries where control activities are strictly based on insecticide-based tools. However, there are some key areas where I would like authors to make clarification/corrections

 Introduction:

- Fourth paragraph, sixth line: it is not clear southern of which country? (Because the previous sentence talks about 3 countries).
- Last paragraph: *Vgsc*-L995F resistance *An. gambiae* Tiassalé strain, correct resistance to resistant

Methods:

- First line: Change senso stricto to sensu stricto. Sixth line: before the word derive write ‘’was’’.

Laboratory resistance selection:

- Here some more details are needed. For the LS group, information is missing on which concentration range was used to find optimal concentration that could results into mortality of 20%, and which concentration were actually picked for selection in the G1, G6, G11 and G16. Also, for the AS group, information is missing on which exposure time range was used to find optimal exposure time that could results into mortality of 20%, and which exposure time were actually picked for selection in the G1, G6, G11 and G16.
- Add information to clarify how selection was done for the LAS group. Example of the two selections, which one did you start with, example: the larvae selection and then waiting for the adult to emerge to do the adult selection.
- Correct the name of a bednet used. In several places you have typed ‘’permanent’’ instead of ‘’PermaNet’’.
- What could be the explanation of progressive increase in mortality against deltamethrin observed in Figure 2 for the NS group?

Discussion:

- Change word ‘’programmers” to “programmes”.

Is the work clearly and accurately presented and does it cite the current literature?

Yes

If applicable, is the statistical analysis and its interpretation appropriate?

Yes

Are all the source data underlying the results available to ensure full reproducibility?

Yes

Is the study design appropriate and is the work technically sound?

Yes

Are the conclusions drawn adequately supported by the results?

Yes

Are sufficient details of methods and analysis provided to allow replication by others?

Partly

Reviewer Expertise:

My area of research is on; efficacy testing of mosquito control tools, mosquito-borne disease control

I confirm that I have read this submission and believe that I have an appropriate level of expertise to confirm that it is of an acceptable scientific standard, however I have significant reservations, as outlined above.

# Response

**Published:** 2023-06-20

**Authors:** France-Paraudie Kouadio

**Affiliations:** Centre Suisse de Recherches Scientifiques en Côte d'Ivoire, Cote d'Ivoire

Introduction:

- Fourth paragraph, sixth line: it is not clear southern of which country? (Because the previous sentence talks about 3 countries). **Done**
- Last paragraph: *Vgsc*-L995F resistance *An. gambiae* Tiassalé strain, correct resistance to resistant **Done**

Methods:

- First line: Change senso stricto to sensu stricto. Sixth line: before the word derive write ‘’was’’. **Done**

Laboratory resistance selection:

- Here some more details are needed. For the LS group, information is missing on which concentration range was used to find optimal concentration that could results into mortality of 20%, and which concentration were actually picked for selection in the G1, G6, G11 and G16. Also, for the AS group, information is missing on which exposure time range was used to find optimal exposure time that could results into mortality of 20%, and which exposure time were actually picked for selection in the G1, G6, G11 and G16.
**The doses chosen to determine optimal concentration are available in the dataset** https://doi.org/10.5281/zenodo.7669512 . **However, we have included the selected doses for regular exposure in the text**.
- Add information to clarify how sele fction was done for the LAS group. Example of the two selections, which one did you start with, example: the larvae selection and then waiting for the adult to emerge to do the adult selection. “Done”
- Correct the name of a bednet used. In several places you have typed ‘’permanent’’ instead of ‘’PermaNet’’. **Done**
- What could be the explanation of progressive increase in mortality against deltamethrin observed in Figure 2 for the NS group?
**The progressive increase in mortality may be explained by the absence of insecticide exposure in the NS group, whereas the opposite was observed in the other groups when mosquitoes were subjected to selection pressure. This suggests that susceptibility can develop in vectors when there is no exposure to insecticides for a long period**.

Discussion:

- Change word ‘’programmers” to “programmes”. **Done**

# Peer review: Reviewer response for version 1

**DOI:** 10.21956/wellcomeopenres.21203.r55594

**Published:** 2023-04-12

**Authors:** Cyrille Ndo

**Affiliations:** 1 Department of Parasitology and Microbiology, Centre for Research in Infectious Diseases (CRID), Yaoundé, Cameroon

**General comment Introduction**

 This is an interesting work which further shows that insecticide exposure at larval and adult stage favours selection of insecticide resistance through elevated expression of detoxification enzymes. This further confirms that management of insecticide resistance could be achieved by rotating the insecticides with different mode of action.

**Recommendation**

 I recommend the paper to be accepted after revisions. Especially, the language and style in the abstract and introduction should be improved.

**Specific comments**

**Abstract**

- Must be improved.

**Introduction**

- To be improved to make it more focused.

**Methods**

**Authors' text:** "Earlier studies have shown that *An. gambiae* s.l from Tiassalé carry the kdr and Ace1 point mutation as well as P450 genes conferring resistance to pyrethroids, DDT, carbamates and organophosphate."

- **Reviewer's comment**: Put “s” at the end of “mutation”.

**Authors' text: **“The mosquito colony used in the present experiments derived from the *An. gambiae* Tiassalé strain maintained in the insectary of Centre Suisse de Recherche Scientifiques en Côte d’Ivoire (CSRS) over several generations since 2010, without being subjected to any insecticide selection pressure.”

- **Reviewer's comment:** This is like a repetition of the first sentence. Please reformulate to avoid repetition.

**Authors' text: **“The experiments used four colonies derived from the same parental population (i.e., G0 parent generation) of *An. gambiae* Tiassalé strain carrying Vgsc-L995F kdr mutation:”

- **Reviewer's comment:** How could this be G0 if the colony has been maintained for long time in the laboratory? This is confusing.

**Authors' text: "**Larvae-elected (LS) group: colony selected with deltamethrin at larval stage over 20 generations"

- **Reviewer's comment:** It is rather “Larvae-Selected”.

**Authors' text: **“Non-Selected (NS) group: colony non-subjected to any insecticide resistance selection, nor at larval stage and neither at adult stage, but reared over 20 generations.”

- **Reviewer's comment:** You can remove “nor at larval stage and neither at adult stage”.

**Authors' text: **“The LS group mosquitoes were selected with a solution of deltamethrin insecticide prepared from a stock solution containing 100mg of deltamethrin (100%) powder in 1000 mL of water.”

- **Reviewer's comment:**
  - Please give the corresponding concentration of the solution of insecticide prepared.
  - Please state why stage II was the only exposed to the insecticide.
  - How was the sub-lethal dose determined? How was the insecticide solution at sub-lethal dose prepared? I think some important details are lacking.

**Authors' text: **”We exposed only stage II larvae of each generation up to 20 generation for 24 hours to a sublethal dose of deltamethrin inducing 20% mortality (LD20). In order to minimize excessive losses while exerting resistance selection, we used the dose causing 20% mortality”

- **Reviewer's comment:** There is a repetition in this sentence regarding the LD20. Please revise it.

**Authors' text: **In the AS group, the selection was conducted by exposing two- to five-dayold, non-blood fed adult female mosquitoes to new PermaNet 2.0 LLIN.

- **Reviewer's comment:** I think it is rather, “five-days old”.

**Authors' text: **"To determine the LT20, we exposed four cohorts of 100 females at different set times in 15×15×15cm holding cages covered with Permanent 2.0 LLIN."

- **Reviewer's comment:** Give details of the set times.

**Authors' text: "**A total of 24 hours after exposure, mosquitoes were blood-fed in order to perform an insectary rearing."

- **Reviewer's comment:** Please reformulate this sentence to make more sense.

**Authors' text: **"To determine the phenotypic resistance within each selected group (i.e., LS, AS and LAS) or non-selected group (i.e., NS), susceptibility tube tests with discriminating doses of deltamethrin (0.05%), bendiocarb (0.1%) and malathion (5%) using treated papers according to WHO protocol7 were performed."

- **Reviewer's comment:** Please indicate if you have treated the papers yourself or if you obtained these from a WHO reference centre.

**Authors' text: "**For each insecticide, four batches of 20–25 two to five-day old female mosquitoes from the three selected groups and non-selected group were exposed to insecticide-impregnated papers in the presence of two negative control batches."

- **Reviewer's comment:** Please describe the negative control.

**Authors' text: **"For each of the four mosquito groups, DNA and RNA extractions were performed with 30 individual mosquitoes randomly selected from the G0, G10 and G20 generations to detect mutation genes whereas for the detection of detoxification enzymes, the target genes expression levels were measured with 50 individuals split into pools of 10 mosquitoes for the G0 and G20 generations only per group (LS, AS, LAS and NS)”

- **Reviewer's comment:** Not sure I understand this. Please reformulate to make more clear. What do the authors mean by mutation genes?

**Authors' text: **“The multiplex TaqMan Real Time – Polymerase Chain Reaction (RT-PCR) methods was used to detect two kdr point mutations from individual mosquitoes and detection of mutations in the voltage-dependent sodium channel target site (i.e., Vgsc-L995F/S) using the protocol developed by Bass et al.30 as adapted by Mavridis et al. 31 (see Table 1 for primers and probes sequences).”

- **Reviewer's comment:** Please reformulate this sentence. It is long and not clear.

**Authors' text: **“We performed the survival analysis to visualize the time of pyrethroid knockdown by comparing the LS, AS, LAS and NS groups to the parent population (G0)”

- **Reviewer's comment:** Not sure I understand what the authors mean.

**Authors' text: **“The expression level of genes determined at G0, G10 and G20 for LS, AS, LAS and NS groups were analyzed using the method of Pfaffl33 implemented the genetic analysis software REST 2009 version v2.013.”

- **Reviewer's comment:** Please revise this sentence.

**Results**

- **Reviewer's comment:** In Table 1 please add a column in which you give references for each primer set. Also provide a legend explaining the meaning of each color in the table.

**Authors' text: **“Overall, very high mortalities (100%) were observed in the LS, AS, LAS and NS groups at any generations (i.e., from G0 to G20) with both bendiocarb and malathion, thus suggesting full susceptibility of all the four mosquito groups to both insecticides (Figure 2A, B, C, D)”

- **Reviewer's comment:** Could be good to rather say “By contrast, very high mortalities (100%) were observed in the LS, AS, LAS and NS groups at any generations (i.e., from G0 to G20) with both bendiocarb and malathion, thus suggesting full susceptibility of all the four mosquito groups to both insecticides (Figure 2A, B, C, D)”

**Discussion and conclusion**

- **Reviewer's comment:** Overall good but more references are needed. Also, the authors could discuss some limits of the study and perspective or significance of their results for vector control.

Is the work clearly and accurately presented and does it cite the current literature?

Partly

If applicable, is the statistical analysis and its interpretation appropriate?

Yes

Are all the source data underlying the results available to ensure full reproducibility?

Yes

Is the study design appropriate and is the work technically sound?

Yes

Are the conclusions drawn adequately supported by the results?

Yes

Are sufficient details of methods and analysis provided to allow replication by others?

Partly

Reviewer Expertise:

Medical entomologist, malaria vector control

I confirm that I have read this submission and believe that I have an appropriate level of expertise to confirm that it is of an acceptable scientific standard, however I have significant reservations, as outlined above.

# Response

**Published:** 2023-06-20

**Authors:** France-Paraudie Kouadio

**Affiliations:** Centre Suisse de Recherches Scientifiques en Côte d'Ivoire, Cote d'Ivoire

**RESPONSE TO REVIEWER REPORTS** **Specific comments** **Abstract**

- Must be improved. **Done**

**Introduction**

- To be improved to make it more focused. **Done**

**Methods**

**Authors' text:** "Earlier studies have shown that *An. gambiae* s.l from Tiassalé carry the kdr and Ace1 point mutation ****as well as P450 genes conferring resistance to pyrethroids, DDT, carbamates and organophosphate."

- **Reviewer's comment**: Put “s” at the end of “mutation”. **Done**

**Authors' text: **“The mosquito colony used in the present experiments derived from the *An. gambiae* Tiassalé strain maintained in the insectary of Centre Suisse de Recherche Scientifiques en Côte d’Ivoire (CSRS) over several generations since 2010, without being subjected to any insecticide selection pressure.”

- **Reviewer's comment:** This is like a repetition of the first sentence. Please reformulate to avoid repetition. **The sentence has been reformulated.**

**Authors' text: **“The experiments used four colonies derived from the same parental population (i.e., G0 parent generation) of *An. gambiae* Tiassalé strain carrying Vgsc-L995F kdr mutation:”

- **Reviewer's comment:** How could this be G0 if the colony has been maintained for long time in the laboratory? This is confusing.

**We considered the parent generation as G0 because it is at this moment that we started the selection.** **Authors' text: "**Larvae-elected (LS) group: colony selected with deltamethrin at larval stage over 20 generations"

- **Reviewer's comment:** It is rather “Larvae-Selected”. **Done**

**Authors' text: **“Non-Selected (NS) group: colony non-subjected to any insecticide resistance selection, nor at larval stage and neither at adult stage, but reared over 20 generations.”

- **Reviewer's comment:** You can remove “nor at larval stage and neither at adult stage”. **Done**

**Authors' text: **“The LS group mosquitoes were selected with a solution of deltamethrin insecticide prepared from a stock solution containing 100mg of deltamethrin (100%) powder in 1000 mL of water.”

- **Reviewer's comment:**
  - Please give the corresponding concentration of the solution of insecticide prepared.

**The corresponding concentration of the solution is 100mg.**L **(10%)**

 - Please state why stage II was the only exposed to the insecticide.

**We have chosen stage II because the larvae are in full growth and it is the appropriate stage to make exposures. Since the exposure is done over 24 hours, if we expose stages III and IV the larvae can pupate during the exposure time, if we also expose stage I they can all die because they are very small. That’s why we exposed only stage II.** - How was the sub-lethal dose determined? How was the insecticide solution at sub-lethal dose prepared? I think some important details are lacking. **We determined the LD _20_ with 100 larvae distributed per batch of 25 larvae and exposed to deltamethrin for 24 hours in plastic cups containing 100 mL of water and fed with 0.075g powdered Friskies cat food. To maintain the selection pressure, a new LD _20_ was determined after every five generations (G1, G6, G11, G16). The corresponding doses determined after every five generations were 5, 6, 14 and 18 mL respectively. The different LD _20_ were determined by counting larvae survival and using PoloPlus 1.0 software (software using probit or logit regression analyses). Prior to each exposure, the 10% deltamethrin solution was prepared 24 hours in advance to allow complete dilution of the deltamethrin powder in water.** **More information are available in the dataset** https://doi.org/10.5281/zenodo.7669512 . **Authors' text: **”We exposed only stage II larvae of each generation up to 20 generation for 24 hours to a sublethal dose of deltamethrin inducing 20% mortality (LD20). In order to minimize excessive losses while exerting resistance selection, we used the dose causing 20% mortality”

- **Reviewer's comment:** There is a repetition in this sentence regarding the LD20. Please revise it. **Done**

**Authors' text: **In the AS group, the selection was conducted by exposing two- to five-day-old, non-blood fed adult female mosquitoes to new PermaNet 2.0 LLIN.

- **Reviewer's comment:** I think it is rather, “five-days old”.

**Adult age was chosen in line with WHO guidance (2016), which recommends the use of mosquitoes aged between two- to five-day-old for insecticide exposure.** **Authors' text: **"To determine the LT20, we exposed four cohorts of 100 females at different set times in 15×15×15cm holding cages covered with Permanent 2.0 LLIN."

- **Reviewer's comment:** Give details of the set times. **Details have been given in the paper.**

**Authors' text: "**A total of 24 hours after exposure, mosquitoes were blood-fed in order to perform an insectary rearing."

- **Reviewer's comment:** Please reformulate this sentence to make more sense. **Done**

**Authors' text: **"To determine the phenotypic resistance within each selected group (i.e., LS, AS and LAS) or non-selected group (i.e., NS), susceptibility tube tests with discriminating doses of deltamethrin (0.05%), bendiocarb (0.1%) and malathion (5%) using treated papers according to WHO protocol7 were performed."

- **Reviewer's comment:** Please indicate if you have treated the papers yourself or if you obtained these from a WHO reference centre. **Done**

 
**Authors' text: "**For each insecticide, four batches of 20–25 two to five-day old female mosquitoes from the three selected groups and non-selected group were exposed to insecticide-impregnated papers in the presence of two negative control batches."

- **Reviewer's comment:** Please describe the negative control. **Described in the text**

**Authors' text: **"For each of the four mosquito groups, DNA and RNA extractions were performed with 30 individual mosquitoes randomly selected from the G0, G10 and G20 generations to detect mutation genes whereas for the detection of detoxification enzymes, the target genes expression levels were measured with 50 individuals split into pools of 10 mosquitoes for the G0 and G20 generations only per group (LS, AS, LAS and NS)”

- **Reviewer's comment:** Not sure I understand this. Please reformulate to make more clear. What do the authors mean by mutation genes? **We mean here kdr mutation genes.**

**Authors' text: **“The multiplex TaqMan Real Time – Polymerase Chain Reaction (RT-PCR) methods was used to detect two kdr point mutations from individual mosquitoes and detection of mutations in the voltage-dependent sodium channel target site (i.e., Vgsc-L995F/S) using the protocol developed by Bass et al.30 as adapted by Mavridis et al. 31 (see Table 1 for primers and probes sequences).”

- **Reviewer's comment:** Please reformulate this sentence. It is long and not clear. **Done**

**Authors' text: **“We performed the survival analysis to visualize the time of pyrethroid knockdown by comparing the LS, AS, LAS and NS groups to the parent population (G0)”

- **Reviewer's comment:** Not sure I understand what the authors mean.

**Normally the survival analysis is used to see the survival time in case of a treatment. However, the scripts also allow to invert the curves in order to observe the mortality by time which corresponds in our case here to the knockdown by time.** **Authors' text: **“The expression level of genes determined at G0, G10 and G20 for LS, AS, LAS and NS groups were analyzed using the method of Pfaffl33 implemented the genetic analysis software REST 2009 version v2.013.”

- **Reviewer's comment:** Please revise this sentence. **Done**

**Results**

- **Reviewer's comment:** In Table 1 please add a column in which you give references for each primer set. Also provide a legend explaining the meaning of each color in the table.

**We have added a column. Each color is described in the first line in assay description column of the table.** **Authors' text: **“Overall, very high mortalities (100%) were observed in the LS, AS, LAS and NS groups at any generations (i.e., from G0 to G20) with both bendiocarb and malathion, thus suggesting full susceptibility of all the four mosquito groups to both insecticides (Figure 2A, B, C, D)”.

- **Reviewer's comment:** Could be good to rather say “By contrast, very high mortalities (100%) were observed in the LS, AS, LAS and NS groups at any generations (i.e., from G0 to G20) with both bendiocarb and malathion, thus suggesting full susceptibility of all the four mosquito groups to both insecticides (Figure 2A, B, C, D)”. **Done**

**Discussion and conclusion**

- **Reviewer's comment:** Overall good but more references are needed. Also, the authors could discuss some limits of the study and perspective or significance of their results for vector control. **Done**
